# Silicon stent placement via rigid bronchoscopy for the treatment of central airway obstruction in infants: Case series

**DOI:** 10.1097/MD.0000000000024244

**Published:** 2021-01-15

**Authors:** Tingting Yu, Le Sun, Xinmei Liu, Wei Zhu

**Affiliations:** Department of Otolaryngology, Head and Neck Surgery, the First Hospital of Jilin University, Changchun, China.

**Keywords:** central airway obstruction, rigid bronchoscopy, silicon stent

## Abstract

**Introduction:**

Rigid bronchoscopy has been proven to be an excellent tool for the diagnosis and management of several causes of central airway obstruction (CAO). The invasive treatment of silicone bronchobrachial stenting has been performed in children and adults with CAO, and satisfying results were obtained in previous studies. However, there are few reports on infants with central airway obstruction treated with stenting via rigid bronchoscopy. This technique remains a challenge to pediatric thoracic surgeons, pediatric interventional pulmonologists, and otolaryngologists who struggle to treat airway obstruction disease.

**Patient concerns:**

Four patients were presented to our hospital with complaints of dyspnea for a period of time after their birth

**Diagnosis:**

Three patients were diagnosed as tracheobronchomalacia, and tracheoesophageal fistula.

**Interventions:**

Four patients were treated with silicone stenting through rigid bronchoscopy.

**Outcomes:**

Silicon stent was adequate for improving the obstruction of the tracheal tract. All the patients were followed-up longer than 6 months. Three patients could breathe normally; the stent migrated in only 1 patient.

**Conclusion:**

Invasive silicone tracheobronchial stenting via rigid bronchoscopy is a viable option for infants with CAO. Choosing an appropriate size is a critical factor for success of stenting according to our experience.

## 1 Introduction

The first rigid bronchoscopy was performed by Dr Gustav Killian in the late 1800 s, and provided physicians with a new glimpse into human anatomy, thereby sparking the growth of pulmonary medicine. Although its application has declined with the introduction of flexible fiberoptic bronchoscopy by Shigeto Ikeda, it is still an invaluable tool in the diagnosis and management of pulmonary diseases. In a 1999 survey, 4% of responders were performing rigid bronchoscopy.^[1]^ Apart from the historic role of rigid bronchoscopy in the treatment of central airway lesions and mechanical debulking of endobronchial lesions, recognition of certain advantages of rigid bronchoscopy over flexible bronchoscopy (such as airway control and ventilation during intervention as well as the ability to simultaneously use larger forceps, suction catheters and tumor excision techniques) has led to an increase in the number of rigid bronchoscopies being performed currently.^[2]^ A multitude of other instruments are now also available to pass down the working channel of rigid scopes, including rigid and flexible suction catheters, different types and sizes of forceps, scissors, rigid and balloon dilators, multiple types of lasers, electrocautery, argon plasma coagulation and cryotherapy catheters, snares, loops, baskets, microdebriders, and stent deployment devices. With advances in flexible bronchoscopy, ablative technologies, and stenting over the past 2 decades, rigid bronchoscopy has become an integral tool in the management of central airway disease.^[2]^ Rigid bronchoscopy remains an important procedure for removing foreign bodies in the trachea.

Central airway obstruction (CAO), although uncommon in children, remains a significant cause of mortality. Thus, treatment is a challenge for clinicians.^[3]^ A number of surgical and non-surgical treatments have been advocated. However, surgeries are difficult for small children with severe airway obstructions and have a high risk of mortality.^[4–6]^ Therefore, a non-invasive approach for addressing this challenge is needed. Endoscopic stenting has proven to be an attractive non-invasive option with satisfactory long-term outcome to manage tracheobronchial obstruction, a potentially fatal condition in infants.^[7]^ Although several kinds of stents (metallic, plastic, silicon) have been used, both advantages and disadvantages of different stents have been reviewed by many clinicians. Silicon stenting is a good choice for stabilization of collapsing airways secondary to tracheobronchial malacia or cartilaginous diseases.^[8]^

Deployment of silicon stents is still based on the original technique described by Dumon in 1990.^[9]^ The role of airway stenting is well-established in adults, whereas the pediatric experience is limited to small series, and is associated with high morbidity and mortality.^[10–13]^ In the case series reported here, rigid bronchoscopy was used to provide operational space and pipeline for stent placement and operation. The aim of this study is to review our experiences with silicone stent placement using rigid bronchoscopy in infants.

## 2 Patient selection and methods

Medical records of 4 pediatric patients who underwent tracheobronchial stenting via rigid bronchoscopy in our Centre were collected. Demographic data, including etiology, associated anomalies, and nature of obstruction were reviewed. Outcome measures included complications such as re-stenosis, granulation tissue, stent migration, fractured stent, maximal achieved tracheal diameter, weaning from the ventilator, and growth at interval follow-up. This study was approved by the ethics committees at Jilin University, and informed parental consent was obtained for each patient (Ethics NO. 2014–336).

**Figure 1:**
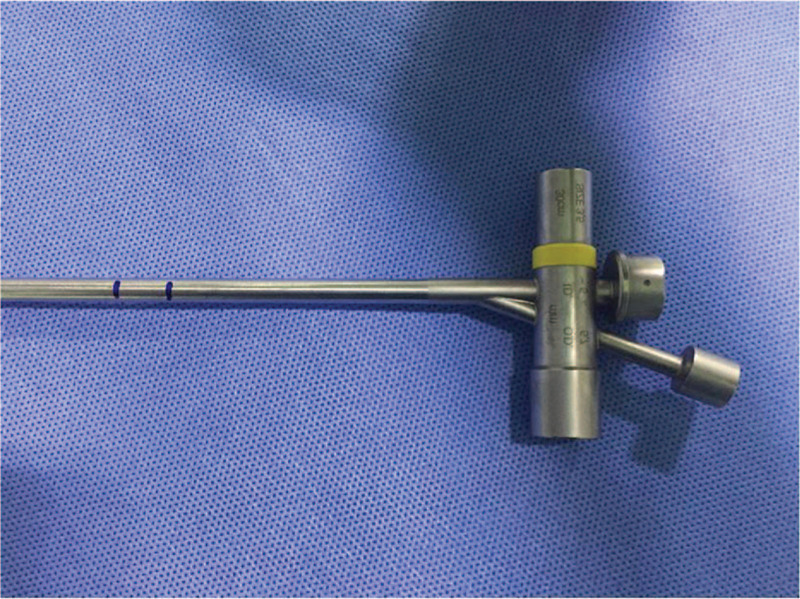
The position marked on a rigid bronchoscope. Before placing the silicon stent, the intubation length was marked on a rigid bronchoscope according to the distance from the incisors to the lesion measured by a flexible bronchoscope.

**Figure 2:**
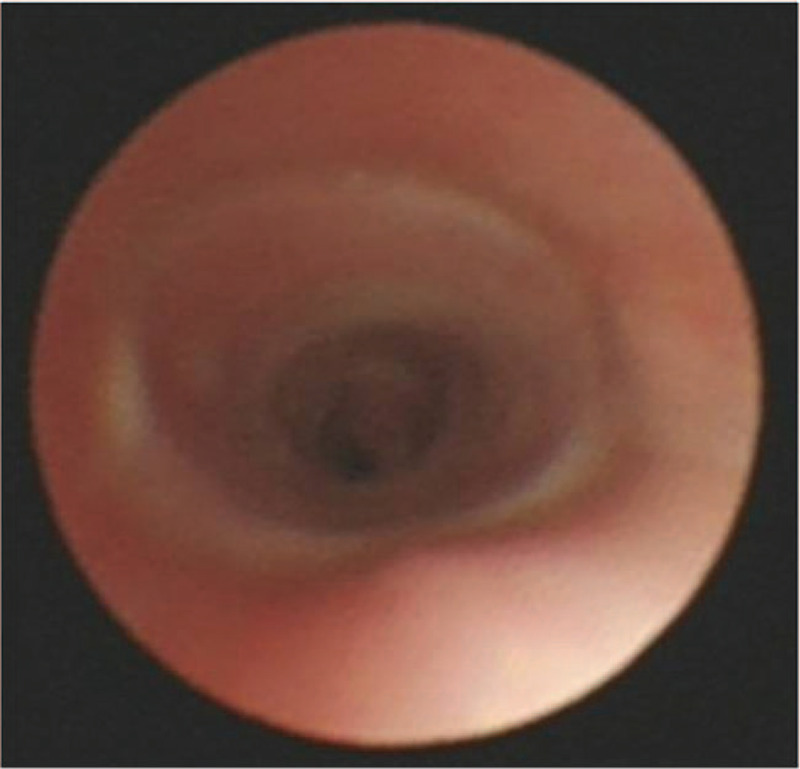
Confirmation of the position by flexible bronchoscopy. After the procedure was completed and the patient recovered from anesthesia, a flexible bronchoscopy exam was performed to confirm the position of the stent. This result showed that the placement site was correct, and there was no edema.

The complete physical assessment and routine blood investigation including hemogram, coagulation profile, renal and liver functions, computed tomography of the chest, and arterial blood gas analysis were done before the procedure. Furthermore, grading of the airway obstruction, a baseline cardiovascular and respiratory evaluation was done to assess fitness. Selection of the stent type depended on the location of the obstruction, the patient's age and body size, as well as the availability of the stent at the time of the procedure. Prior to stent placement, the location, severity, and length of the obstruction were assessed by pediatric respiratory physicians via flexible bronchoscopy (Olympus BF P260F, Tokyo, Japan). In all 4 patients, silicon stents were placed using a Storz rigid pediatric bronchoscope (Karl Storz, Germany) under general anesthesia. The stenting site was determined by simultaneous detection, marking the distance from the lesion to the incisors during the rigid bronchoscopy procedure (Fig. 1). The frizzled stent (DUMON) was placed at the terminal position of the scope and pushed down into the tracheal canal to the marked position. The position of the stent was again confirmed, and the tracheal mucosa was visualized by flexible bronchoscopy (Fig. 2).

## 3 Results

**Figure 3:**
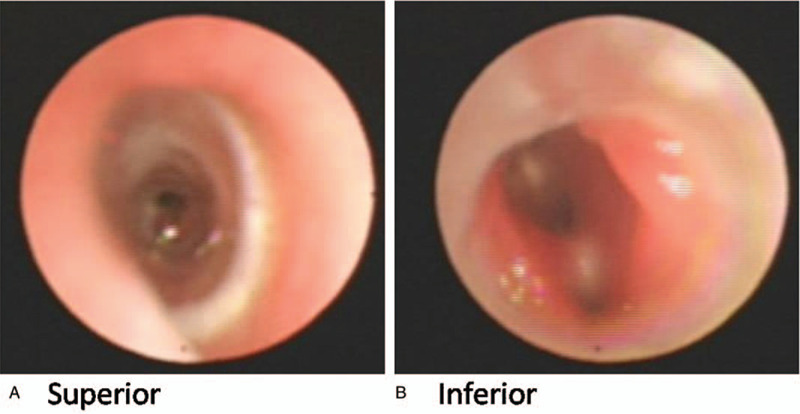
Follow up after 1 month. A. The tissues superior to the silicon stent were normal, and the position of the stent was stable. B. Some granulations were found at the inferior part of silicon stent. However, the tracheal cavity was unobstructed. There was no dyspnea.

**Figure 4:**
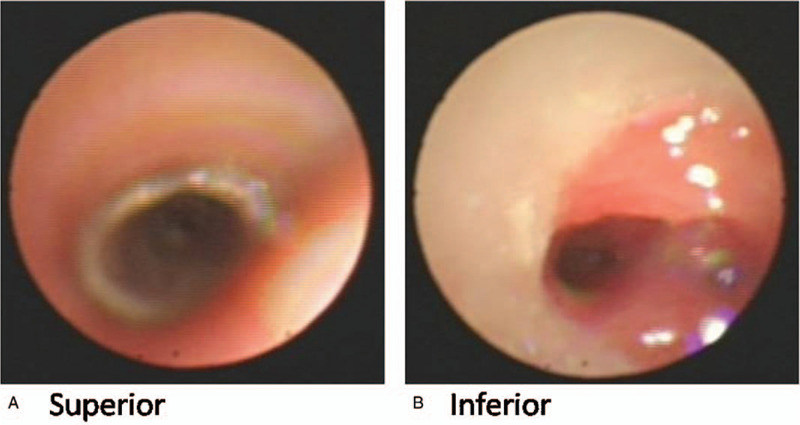
Follow up after 4 months. A. The tissues superior to the silicon stent were normal, and the position of the stent remained stable. B. Granulations were increased 1 month after stenting at the inferior part of silicon stent. However, the tracheal cavity remained unobstructed. There was no dyspnea.

**Figure 5:**
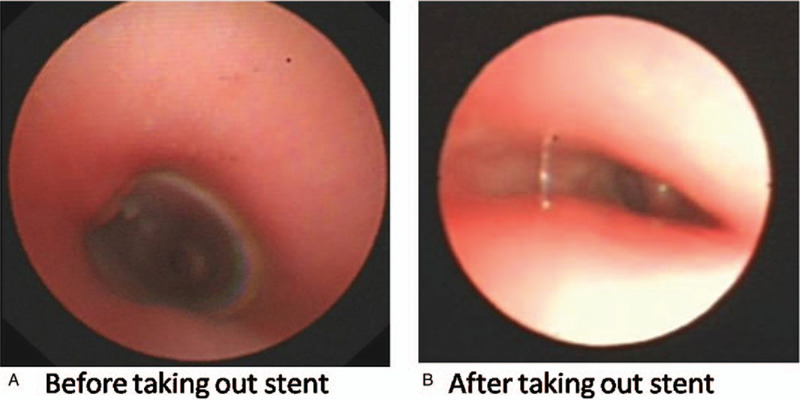
Follow up after 6 months. A. The tissues superior to the silicon stent remained normal. B. The silicon stent was removed from the trachea, stenosis was still present. However, the symptom of dyspnea was alleviated significantly.

Four patients (1 girl, 3 boys) presented with respiratory distress during the neonatal or early infantile period, and all were referred to the pediatric respiratory department of our hospital for further treatment due to dyspnea and chronic cough. Three were diagnosed with tracheomalacia; the other one was diagnosed with tracheoesophageal fistula. Surgeries were offered just after birth to the patients diagnosed with tracheoesophageal fistula, but the surgery was not successful. Patient demographics, the anatomy of tracheobronchial obstruction, location of the stents and complications are reviewed in Table 1. The mean age for stenting was 7 months (range: 3–9 months). One patient (case 3) with severe pneumonia due to a tracheoesophageal fistula underwent stent removal just 20 days later due to unsatisfactory improvement. All patients were followed up closely, and there was no peri-operative mortality. We found that the silicon stent played a very good stenting function for the tracheal tract. All the patients except Patient 3 had normal breathing after stent placement. For case 4, the images obtained by flexible bronchoscopy at 1, 4, and 6 months after stent placement are shown in Figs. 3–5, respectively. Some granulation tissue was observed at the inferior margin of the stent. However, its proliferation decreased subsequently. The stent was removed from the trachea via rigid bronchoscopy 6 months after placement. Although the trachea was still a little narrow, the patient could breathe normally and in a calm manner without any auxiliary equipment.

**Table 1:** Patient demographics, stent location, and complications.

Patient	Sex	Age at stenting (mo)	Primary disease	Anatomy of the fistula/malacic segment	Location of stent	Complications at stent removal
Case 1	Boy	7	Tracheobronchomalacia	15 mm segment of tracheal malacia 15 mm above the carina with complete cartilage rings	1 in distal trachea	Nil
			Laryngomalacia			
Case 2	Boy	8	Tracheoesophageal fistula	22 mm segment of tracheal malacia 18 mm above the carina and sutures could be seen at 28 mm above the carina	1 in proximal trachea	
			Tracheobronchomalacia			
Case 3	Boy	3	Tracheoesophageal fistula	20 mm segment of tracheal malacia with bilateral fistulas 18 mm above the carina	1 in proximal trachea	Migration
Case 4	Girl	9	Tracheobronchomalacia	15 mm segment of tracheal malacia 15 mm above the carina with complete cartilage rings	1 in proximal trachea	little granulation

### 3.1 Case 1

As a result of congenital patent ductus arterious, this male infant required an operation of closure at the age of 5 months. He suffered from pulmonary infection frequently due to laryngomalacia and tracheobronchomalacia. The flexible bronchoscopy showed that the lumen 15 mm above carina of trachea collapsed on inspiration. A 15∗7 mm silicone stent was placed in the lesion of trachea to resolve the dyspnea. There was no complication during the placement procedure. He was followed up every month regularly. There was no severe dyspnea, and some small granulation above the stent was observed through flexible bronchoscopy till the stent was removed 5 months later.

### 3.2 Case 2

This male infant was diagnosed as congenital trachoesophageal fistula (TEF) and patent foramen ovale when he was 3 months old. He underwent tracheoesophageal fistula neoplasty. At 8 months of age, he presented to our hospital with severe pneumonia. Flexible bronchoscopy showed that the membrane portion of trachea was enlarged and the lumen was narrow. The suture was seen on the inferior part of the collapsed trachea. However, there was no gastric fluid leakage from esophagus. For enlarging the stenosis of trachea, a 20∗7 mm silicone stent was placed 18 mm above the carina. He was followed up each month. Evaluation revealed good breath, no stridor and some granulation was observed at the distal interface between the stent and the trachea at the level above carina from 2 months till the 6^th^ month (at the time of writing this manuscript).

### 3.3 Case 3

Congenital esophageal atresia (CEA) and TEF was noted in this male infant immediately after delivery. Anastomosis of esophagus and tracheoesophageal fistula neoplasty were performed in pediatric surgery of our hospital. Although initially, the patient was well, he had repeat life-threatening apneic episodes as a result of severe tracheomalacia between 2 and 3 months of age. Endoscopy and CT scan corroborated the stenosis of trachea and the recurrence of TEF. A 20∗5 mm silicone stent was inserted and the dyspnea ceased. However, on the 5^th^ days after stenting, endoscopic evaluation showed that the stent had moved upward to the subglottic portion. It pushed into the trachea again and the fistula was covered. For the next 20 days, he showed good breathing with good feeding. However, the parents requested to remove the stent for further surgery of TEF in another hospital.

### 3.4 Case 4

This term male infant was born with CEA, severe respiratory distress, acute cardiac injury. He required an anastomosis of esophagus and tracheoesophageal fistula neoplasty at 2 days of age. At the 9 months of age, he was noted to have intermittent episodes of tachypnea. Evaluation by flexible brochoscopy showed the stenosis of middle and lower part of trachea. A fissure was also seen 20 mm above the carina. A 20∗7 mm silicone stent was inserted into the stenosis part. There was no complication during the follow-up. Mild granulation was found at the distal interface of the stent through flexible bronchoscopy till the stent was removed out 6 months later.

## 4 Discussion

A number of retrospective studies have reviewed complications following bronchoscopic treatment of CAO. A recent multicenter registry trial published by Ost et al examining 1,115 procedures performed on 947 patients at 15 centers from 2009 to 2013 showed only 44 patients (3.9%) reported complications.^[14]^ However, a multitude of risks related to rigid bronchoscopy and silicone stent placement should be considered, including trauma to the oral cavity (lips, gums, teeth, tongue, pharynx), the vocal cords, the trachea and bronchi, bleeding, infection, hypoxia, respiratory failure requiring mechanical ventilation, tracheostomy, cardiac arrest, and death. Metallic stenting via brochoscopy may cause stent damage, fracture, or displacement.^[11]^ According to our experience, the silicone stent is rarely damaged during the manipulation procedure.

Notably, a full history is essential to establish the reason for stent insertion, and its position and size. Sizing is extremely important, as proper sizing decreases the chance of migration and the formation of granulation tissue, airway fistulation, misplacement, or difficulty in deployment. Chest computed tomography is one way of visualizing the stent position, and measurement during bronchoscopy is another option.^[15]^ We prefer to determine the stent diameter during the flexible bronchoscopy procedure as we can observe the altered respiratory canal clearly during breathing.

Deployment of the silicone stent is according to the technique previously described by Dumon. The appropriate frizzled stent was manually loaded into the distal side of the bronchoscope. The stent was then pushed down to the trachea by a prosthesis pusher. This procedure is difficult in infants due to the high position and narrow glottis crack. The risk of laryngeal edema is increased significantly by repeated bronchoscope intubations. It was difficult to visualize the integral glottis crack in our first patient, but we finally succeeded after 4 attempts. This difficulty resulted not only from the high position, but also the malacic epiglottis cartilage. In addition, if the stent does not fully open, specialized equipment should be used to open the stent carefully without pushing it distally or destroying the tracheal mucosa.^[9]^ Airway stenting is also performed in combination with surgery for the treatment of severe bronchomalacia, or prevention of post-tracheoplasty restenosis.^[16–18]^ In our study, 2 cases were diagnosed as esophageal fistula, 1 was a post-surgical esophageal repair, and the lesion sites could be seen when placing the bronchoscope and stent. A proficient technique for this procedure was needed to obviate complications from misplacement and additional injury. Crucial to the management of this condition is delicate manipulation and careful guidance during visualization. One case experienced migration when examined by flexible bronchoscopy on the first day after insertion of the stent, and the stent was adjusted via flexible bronchoscopy guidance. However, the outcome of ventilation was unsatisfactory, and the stent was removed after twenty days.

Silicone stents may be only temporarily effective in infants. A new, wider stent may be substituted, or other medical treatments, such as invasive surgical options, may be required when the inserted silicone stent fails. Besides the surgical procedure, anesthesia and the post-stenting complications should also be considered. Cooperation and communication between the surgeon and the anesthesiologist are required to ensure patient safety. Formation of granulation tissue is a common complication post-stenting. Granulomatous reactions are infrequent and moderate in patients with silicone stents,^[19]^ in line with our experience. In the follow-up images obtained via flexible bronchoscopy in one of our patients, some granulation tissue was seen at the inferior end of the silicone stent. However, it did not influence the patient's respiratory function. She had a good sleep and normal capacity to suck milk.

Although either silicone or metallic tracheobronchial stenting successfully resolves the CAO in infants in earlier reports, they are still not the “ideal” airway stent, for each one has its own advantages and disadvantages. Biodegradable stents have been offered as a new stent type which is safe, effective, and causes fewer complications.^[20]^ However, more research is needed to fully characterize these new “ideal” stents and devices.

In our case series, good outcomes were achieved in most patients. And till now, there was no report on stenting for infants who are younger than our cases. However, the follow-up period was short, the longest being 2 years. In addition, the number of cases was small. We intend to improve our skill to decrease the operative time and reduce the risks associated with the procedure.

## 5 Conclusions

Invasive silicone tracheobronchial stenting via rigid bronchoscopy is a viable option for infants with CAO, but it should be performed only by expert otolaryngologists because of the technical difficulties involved. It is an important technical skill for otolaryngologists as it can relieve airway obstruction in infants with CAO, and is associated with only moderate complications.

## Author contributions

**Data curation:** Le Sun, Xinmei Liu.

**Writing – original draft:** Tingting Yu.

**Writing – review & editing:** Wei Zhu.

## Abbreviations

Abbreviations: CAO = central airway obstruction, CEA = Congenital esophageal atresia, TEF = trachoesophageal fistula.
